# Health and Social Care Inequalities: The Impact of COVID-19 on People Experiencing Homelessness in Brazil

**DOI:** 10.3390/ijerph18115545

**Published:** 2021-05-22

**Authors:** Nilza Rogéria de Andrade Nunes, Andréa Rodriguez, Giovanna Bueno Cinacchi

**Affiliations:** 1 Social Work Department, Catholic University of Rio de Janeiro, Marquês de São Vicente Street, 225, Rio de Janeiro 22451-041, Brazil; 2 School of Dentistry, University of Dundee, Park Place, Dundee DD1 4 HN, UK; a.rodriguez@dundee.ac.uk; 3 Social Work School, Campus do Gragoatá, Federal Fluminense University, Marcos Waldemar de Freitas Reis Street, Bloco E, Niterói 24210-201, Brazil; giovannacinacchi@gmail.com

**Keywords:** homelessness, COVID-19, health inequalities, social care, participatory research, Brazil

## Abstract

This article aims to reflect on the challenges affecting people experiencing homelessness in Rio de Janeiro, Brazil, due the COVID-19 pandemic. Participatory research was carried out to identify data related to sociodemographic profile; strategies for survival; health and social care support; and access to services during the pandemic. The research methodology was co-designed with NGOs and people with lived experience of homelessness and involved conducting semi-structured questionnaires with 304 participants in 2020. The results highlighted the worsening of the situation of extreme vulnerability and poverty already experienced by this population before the pandemic. Key strategies led by Third Sector organizations to reduce the spread of the virus, to minimize the financial impact of lockdown, and to increase emotional support and information on COVID-19 were presented. The conclusions show the complexity of issues affecting these groups and the need for urgent response from public policies and Government support to guarantee their rights, dignity, and respect during and after the COVID-19 pandemic.

## 1. Introduction

The world is experiencing the greatest global health and humanitarian emergency of the century, considering that we have already accounted for more than three million lives lost worldwide caused by the SARS-Cov-2 coronavirus. In Brazil, the first case was reported on 26 February 2020. The first cases of contagion occurred in the upper classes who came from trips to Europe, which was then impacted by high contamination rates and deaths. However, the first death reported in Brazil was of a domestic worker contaminated by the employers who were infected when they returned from a trip to Italy. During the first weeks, most of the reported deaths were of white people from more privileged and affluent classes. Over time, the Brazilian social and racial inequality was evidenced by the expressive increase in the number of deaths among the black and peripheral population.

Regarding the COVID-19 pandemic situation in 2021, Brazil is in second place on the global scale in the number of deaths and infected people, with 3251 deaths in a single day in March 2021, according to the Ministry of Health. In the same period, more than 12 million people were infected and there were over 420,000 deaths [1]. A Unifying Panel of COVID-19 in Rio de Janeiro monitoring deaths in favelas showed that there were 3396 deaths in the first three months of 2021 in these territories, accounting for more deaths than in 162 countries.

Data show that the deaths by COVID-19 not only emphasized the great existing inequalities and inequities in the country but also deepened them. The adverse conditions of precariousness and exclusion of certain social groups before the pandemic were especially affected by factors such as informal employment, lack of social protection, and limited access to health services, emphasizing the social conditions that generate and perpetuate situations of vulnerability, resulting in obstacles and unwanted effects experienced by the population when adopting and complying with public health measures.

Studies revealed that most of the homeless population has difficulty accessing health services, especially preventive ones [2,3,4]. This represents a strong obstacle to promote health to these groups, which is even worse in a pandemic context that contributes to the emergence of serious diseases and premature death [5,6]. Despite the implementation of specific public health programs and policies for this population, which precedes a pandemic, the lack of information, the stigma, racial, gender, and sexual orientation discrimination are still factors that continue to hinder access to services and the health care of people experiencing homelessness in Brazil [7].

The existing services do not address the real needs and demands of this population. Some authors consider it urgent that professionals working in public health services receive specific training to treat this population, since there is a lack of deep knowledge of their life contexts and their particularities [8]. The result of this lack of investment in regular professional training often leads to homogenized and stigmatized practices of care and social assistance in the view of this population, which does not help them to get out of the situation, and the cycles of poverty and homelessness persist [9]. Studies point out that access to a greater offer of services to the homeless population, occurs, preferably, through urgent, emergency, and crisis services [10,11]. However, as much as these services guarantee the first care, the continuity of follow-up/care remains a challenge to be overcome by professionals, services, and public policies.

In Brazil, the health crisis caused by the COVID-19 pandemic coexists with a major crisis, which is ethical, economic, social, and political. Some measures adopted by the Federal Government to fight the pandemic such as the encouragement to use alternative medicines without scientific evidence as “early treatment”, the denial of the need to follow social isolation to avoid the detriment of the economy, the public politicians’ statements questioning the use of masks and the effectiveness of vaccines, among others, went against all recommendations from national and international health authorities. There are flaws in the national response plan and the logistics of vaccination in Brazil, with strong scientific denialism [12]. While several countries have expanded investments in social policies to mitigate the impacts of the pandemic on the poor population, the Brazilian Government chose to strengthen economic policies, exposing a huge number of workers to pandemic conditions, which also includes starvation and misery.

Understanding the necropolitics of Achille Mbembe [13] helps us think about what is happening in Brazil [12]. This concept describes the power to determine who can live and who should die. Death becomes acceptable, but not for all people and bodies. The “killable” body is always the one defined by the race parameter, which reflects on the poorest and blackest [13].

Among the most vulnerable social groups affected to this health, social, and economic crisis, we highlighted the homeless population. Studies on the homeless population in Brazil and the effects of the pandemic are relatively scarce and need to be better developed [14,15]. To the extent that authorities in much of the world recommend to “stay home” and the adoption of restrictive measures of mobility and social interaction, these actions are inaccessible to people living on the streets in Brazil. Preventive measures against COVID-19 such as social isolation and constant hand hygiene are much more complex to be taken when there is no home or when it is crowded and inappropriate for human beings. In Brazil, this population is defined in the National Policy for the Homeless Population, through the Decree 7053/2009. The population is defined as, “a heterogeneous population group having in common extreme poverty, interrupted, or weakened family ties, and lack of regular conventional housing, thus using public places and degraded areas as temporary or permanent living spaces, as well as shelters for temporary overnight stays or as temporary housing.” [16].

In addition to the lack of housing and the use of the streets as a space for survival, some particularities tend to be common to individuals facing this problem. Characteristics such as gender, race/ethnicity, age group, nationality, and level of education indicate a certain level of homogeneity of this public. The vast majority of those living on the streets are men (82%), black or brown, aged between 18 and 35 years [16].

The female population represents the other 18% of the total amount of people living on the streets. Most women are also young, and they go to the streets at an earlier age than men: 21.17% of them are between 18 and 25 years old, and 31.06% are between 26 and 35 years old. The streets are fertile scenarios that express these social exclusion, and their invisibility takes the place of the (in)existing ones. Difficulties in accessing social policies, especially health care and social protection, are insufficient to reach this social segment, which increasingly expands in numbers—a cruel reality.

In Brazil, the homeless population is not considered in census surveys of the Brazilian Institute of Geography and Statistics (IBGE). The first and only national survey that considered people experiencing homelessness was carried out in 2008 by the Ministry of Social Development [7]. However, some Brazilian municipalities carry out their own census surveys, such as Rio de Janeiro. In 2018, a survey by the Municipal Secretariat of Social Assistance and Human Rights of Rio de Janeiro stated that 4628 people were living on the streets at the time. This survey was criticized by several sectors because according to the latest data of this secretariat, in 2016, the city had three times more homeless people than this (14,729) [17]. In 2020, a new Census of the Homeless Population was carried out in the city of Rio de Janeiro indicating a total number of 7272 people, but the data were only officially released in 2021. Questionnaires were applied on the street, in crack use scenarios, in homeless shelters, and in therapeutic communities [18]. However, the number differs considerably from those disclosed by the Forum of Adult Homeless Population in the State of Rio de Janeiro and the Public Defender’s Office of The State of Rio de Janeiro, which suggests a population of about 15,000 people.

Victims of social invisibility and with multiple social determinations for health linked to their life stories, the homeless population bears the mark of a marginalized society, suffering with stigma, discrimination, and prejudice manifested in a state of injustice and violence [19]. The public policy of social assistance to this group in the context of Rio de Janeiro has only two Specialized Reference Centers to care for the adult homeless population. This is a service within the scope of the Special Social Protection of Medium Complexity at the Unified Social Assistance System, providing socio-assistance services in the context of education, territoriality, and specialized support during day and night. In the field of health, the care approach takes place through seven Consultório na Rua teams (outreach services) spread throughout the city, but the lack of staff and structure resources compromise and limit their capacity of care.

Social isolation for those who have the streets as their home, hand washing for those who do not have regular access to clean water, protection for hands, mouth, nose, and eyes for those who often have garbage sale as a source of income and survival are some of the challenges that have become even more explicit in this pandemic. This population group cannot follow the guidelines described due to the living conditions in which they find themselves.

The homeless population is continuously exposed to the lack of public policies to ensure them housing, health, education, and care, among other rights. In pandemic times, they are in agony. In 16 March 2020, as restrictive measures to fight contagion were implemented in Rio de Janeiro, this resulted in closed commercial establishments and low circulation of people on the streets. Therefore, the few sources of income that this population has (while working with recycling, selling candies and sweets at the traffic lights, begging for money, among others) were reduced. However, hunger does not end, and the sanitary conditions required as a preventive measure became impossible to be fulfilled, as recommended by the World Health Organization.

Given the invisibility of this population group, a survey was carried out from August to October 2020 in Rio de Janeiro that sought to map the social and health conditions worsened by the pandemic. There was no specific protection plan for this segment from the Government to ensure their food security, basic sanitary conditions, emergency assistance, and access to health and social care services considering the specificities required by these groups, besides the limited offer of interventions with a person-centered and humanized approach.

## 2. Materials and Methods

The study used a participatory research methodology with strong involvement of organizations from the Third Sector working with people experiencing homelessness in the city of Rio de Janeiro. Previous work [11] on a knowledge exchange program on homelessness and accessibility of services developed in Scotland and Brazil had shown the need for further studies on the Brazil case, especially during the pandemic period.

The research methodology was co-designed by the Working Group consisting of PUC-Rio University (the Department of Social Work), University of Dundee/UK, and two NGOs: Porto ComVida, founded at the beginning of the pandemic, and Pastoral do Povo da Rua, an organization with decades of experience working with homeless people. The project was submitted and approved by the Ethics Committee of PUC-Rio University (PUC-Rio 006/2020—Protocol 045-2020).

The combination of experience from partner organizations in providing research and direct intervention in this field and the international collaboration of University of Dundee was essential to reach this public during the pandemic and to produce data on their experiences and challenges to mitigate these impacts.

The study was based on a survey with a simple random sample carried out in the intercept format with the research participants from August to October 2020. It was also based on the universe of 3200 people experiencing homelessness who received weekly food provision in 23 areas of the city. It was intended to reach a total of 395 semi-structured questionnaires that represented a 95% confidence level and 5% margin of error. However, due to the difficulties related to using the internet, sun light (in some cases, the distribution of food occurred at evenings/nights), and participants under the influence of alcohol and other drugs, 304 interviews were obtained (259 homes and 45 women). This amount represents a small increase in the margin of error, from 5% to 5.3%, which does not preclude the results obtained in the survey. This sample used the formula

(1) $$n=\frac{N\sigma {\;}_{\;\;\;\;zy}^{2}∕2^{2}}{\left(N-1\right)\varepsilon^{2}+\sigma^{2}z_{y}∕2^{2}}$$

for calculating finite population with less than 100,000 inhabitants.

The recruitment process of research participants was supported by 30 volunteers working in the NGOs’ partners. Their selection was based on their great knowledge on homelessness and familiarity with the territory, with some of them presenting lived experience on homelessness as well.

During the delivery of food/meals in these 23 mapped points with high concentration of rough sleepers (city center, parks, areas in front of churches, shops, restaurants), the team of volunteers from the two NGOs partners approached users and explained the research aims and consent form. The questionnaires were administered to those who agreed to participate. There were no incentives for research participants, and they were chosen at random while arriving at one of the 23 points of food distribution. Usually, the questionnaires were conducted by a pair of volunteers that tried to identify a reserved place as much as possible for participants to respond the questions, before or after the receiving of food/meals.

The instrument used for data collection was an electronic questionnaire (made at the Google Forms platform) composed of 29 closed multiple choice and open questions that were filled out by these 30 NGO volunteers with the general coordination of researchers from PUC University. The questionnaire was co-designed with the participation of these volunteers to allow the use of adequate language and easy understanding for the public involved. The questionnaire covered key themes: sociodemographic profile; street life and survival strategies; approaches and access to service and COVID-19.

The group of researchers and NGO volunteers was trained in participatory research and counted on frequent supervision of the research coordination team. All safety protocols related to COVID-19 were followed during data collection, such as social distancing, use of mask, and alcohol gel. The data were recorded in a database in the Statistical Package for the Social Sciences (SPSS).

## 3. Results

### 3.1. Sociodemographic and Economic Profile

The total of 304 research participants presented a demographic profile that seems to be representative of people who can be categorized as homeless, since they were similar in their demographic profile to other homeless populations living in other cities in Brazil [20,21]. Age, race, education, income-generating activities, health status, street life-related attitudes and behaviors were also similar to other populations experiencing homelessness across Brazil [22].

The survey pointed out the predominance of male gender, with 85.2% of the total, 72.7% of whom were black and brown. Regarding the age group, the study showed that 42.1% were between 41 and 59 years old, followed by 32.6% aged between 31 and 40 years old. Our research did not find a high incidence of individuals of other nationalities, with only two cases of migrants.

Regarding the formal educational level, there is a predominance of low education. The most predominant education level corresponds to Incomplete Primary School with 42.8% of individuals, followed by Complete Elementary School (18.8%), and Incomplete High School (17.1%). Less than one percent of our audience had completed Higher Education. Table 1 shows the sociodemographic profile of the participants of our research. The variables correspond to the different analysis categories. 

Differences in data from men and women experiencing homelessness were noticed. Women stated more health problems before the pandemic (43% women against 24% for men). They also had more levels of unemployment (20% of women without any source of income and 7% for men in the same position). However, women presented high levels of education when compared to men (25% for women with high school degree and 16% of men).

### 3.2. Access to Services

Participants were asked about the offer and access to health and social services before and during the COVID-19 pandemic. Questions about personal hygiene and treatment when having a health problem were made. Participants mentioned been approached by one or more services. The services that most appear to be accessed before and during the pandemic were from social care sectors (56.3%). Almost one-quarter of the sample (23.7%) said they had been approached by public security agencies. We know that this public is a vulnerable group suffering vexing and violent practices from the military police that aimed for “social cleaning”.

Actions from the military police are often required from inhabitants living or working near territories occupied by rough sleepers to remove them, as discrimination and stigma against these groups are common in Brazil. “The presence of undesirable people appears as a reason for vacating the streets and reducing economic activities.” [23].

Only 15.5% of the respondents stated that they had been approached by health care services such as *Consultório na Rua* (outreach teams), which is a specific service for this population. This service is configured as a strategy from the National Primary Care Policy and states that health care for this specific population should be the responsibility of all health professionals in the Brazilian Unified Health System (SUS), especially in Primary Care, as it is to any other citizen [24].

The teams of *Consultório na Rua* “comprise health professionals with the responsibility of articulating and providing comprehensive health care to homeless people” [24] (p. 62). As structural features of the public health policy, *Consultório na Rua* should deliver comprehensive care, intersectoral articulation, a biopsychosocial approach, and activities of harm reduction. However, there are only seven pieces of equipment distributed in the city of Rio de Janeiro, and none in the south area, which is not enough to meet the real demands. Multi-professional teams for this service can be formed by three modalities. The first comprises a nurse, psychologist, social worker, and occupational therapist. The second has a social agent, nursing technician or assistant, dental health technician, dental surgeon, physical education professional, and an art-education professional. The third modality is similar to the first one but has the addition of a medical professional. The key aims are the development of comprehensive health care for this population, carrying out their activities in an itinerant manner, and whenever necessary develop actions in partnership with teams from the Basic Health Units in the territory of Rio de Janeiro [25].

However, the small percentage of participants who reported having been assisted by Consultório na Rua may be represent a direct result of the huge health system crisis that Brazil is facing with the current President of the Republic. Under this Government, public spending on health and education was controlled and frozen for twenty years. Several health services for the poorest populations and those living in slums (favelas) and peripheries have suffered from budget cuts, lack of professionals, and even services closure due to the lack of basic equipment and structure to work.

The Public Defender’s Office was another service mentioned by participants with a lower percentage of approach to these groups. In Rio de Janeiro city, legal actions toward homeless population are carried out by the Center for the Defense of Human Rights of the Public Defender’s Office of the State of Rio de Janeiro. The general misinformation of this population about the existing services, their rights, as well as the lack of address, documentation, low level of education (required to fill out registrations and schedules), along with the stigma and discrimination felt when accessing basic services have a strong impact on their feeling of inadequacy and distance from services.

Some people reported not having been approached by any service (18.8%) during their life on the streets, which is not surprising considering the context of huge social exclusion and health inequalities in Brazil. The precarious situation of public services in Brazil is not recent and dates to a history of lack of federal investments in public health, education, and social assistance facilities. The invisibility of these groups in society regarding the defense of their rights as well the developing of public policies means that homeless people are only visible to society when they become “a discomfort”.

A significant number of respondents (80.3%) pointed out the use of public services to address health issues. However, they only accessed the services when in serious health conditions, great pain, and/or when the problem affected their ability to survive on the streets. There are barriers to access health services, such as stigma and discrimination from practitioners. A rough sleeper often needs to be accompanied and monitored by family members, friends and front-line staff to ensure that the provision of care will be guaranteed [26].

A percentage of 31.6% participants considered the Basic Health Clinics and the Family Health Clinics to be the best equipment on health, and 29.9% rated the Emergency Care Units as being the best one, which was followed by 8.9% mentioning the hospitals and 7.6% mentioning the *Consultórios na Rua*, despite this being a specific and qualified service created by a health policy to serve this public. Family Health Clinics are a model of Primary Health Care in the city of Rio de Janeiro aimed to focus on prevention, health promotion, and early diagnosis of diseases. The Basic Health units would be able to solve 69.1% of cases of those searching for care. The emergency health care units meet the intermediate complexity and provide 24 h service. Table 2 shows the access to services by the research participants. The variables correspond to the different analysis categories.

### 3.3. Impact of COVID-19 and Homeless People

The study aimed to explore if COVID-19 had brought new challenges and changes in the daily lives of those experiencing homelessness, such as changes in accessing services, hygiene products to mitigate the spread of the virus and/or any other extra support. Participants were asked if they felt symptoms of COVID-19 and other pre-existing diseases.

The hygiene of hands during the pandemic was one of the main pieces of advice to avoid COVID-19 transmission according to health authorities, and at a time when media campaigns were saying to “wash your hands well”, this group was experiencing an extreme disadvantage in relation with people not experiencing homelessness. The homeless population in Brazil is mostly composed for rough sleepers. Actions from civil society organizations regarding facilitating the access to hygiene products for these groups were essential. A social project called ‘Pia do Bem’ (good sink) installed more than one hundred mobile sinks in the municipality and the Metropolitan Region of Rio de Janeiro in April 2020 to help with the hygiene maintaining of this population.

The impacts of the pandemic are much more intense on vulnerable groups such as those experiencing homelessness as the stores/shops closed and emptied the streets. Access to financial income for a large part of this population occurs through precarious work (recycling, selling products on the streets, cleaning jobs, construction, and by begging for money and food in front of restaurants, stores, and cash machines). These activities were dependent on the movement of the cities, and with empty streets, it became extremely difficult for this group to earn money even to buy food.

Thus, the food security of this group was quickly threatened. Our data showed the importance of civil society support in feeding these people. When asked what kind of extra help they were receiving during the pandemic, 83.2% said they were receiving food from NGOs (meals or breakfast), and 10.2% said they received basic food baskets. Meals were considered more appropriate to them due to difficulties in cooking on the streets.

Another piece of relevant data was the distribution of items of hygiene. More than half of participants said they received this type of product coming from non-governmental organizations as an extra help during the pandemic. Conversation and emotional support were mentioned by 19.7% of people. Such data are relevant, since there is a tendency to exhaustion and mental suffering arising from these groups with the pandemic situation caused by COVID-19.

The federal government provides social benefits for the poor population such as the Bolsa Família Program (for those without work and with children in school age), and for old people aged 65 years and over who did not contribute to social security. An extra emergency support aid of R$ 600.00 (the equivalent of 85£) was provided to address the impacts of COVID-19 from April 2020 to January 2021. Only 4.9% of the survey had accessed one of these benefits. The barriers for accessing benefits (lack of documentation, absence of services in the territory they live, lack of knowledge, reduction of actions on the street due to the pandemic) as well as the low offer of services and their precarious conditions can explain this data. Clothing and medicine had a 1.6% incidence on the results, and only 1.6% reported not receiving any extra help.

When asked if they had pre-existing health conditions 26.3% of individuals responded positively. Those who said they did not have any disease or comorbidity constituted 66.8%, and those who did not know made up 5.9%. For those who responded positively, an open question was asked about what diseases they had, and more than one option could be mentioned. In the group with pre-existing diseases, 27.5% mentioned they had a heart problem, with the highest reports being arterial hypertension. The second highest health issue was respiratory problems (tuberculosis, bronchitis, pneumonia, sinusitis, or the flu), with 22.5% of the total of the sample. About 17.5% percent of respondents reported having HIV, with only one case of coinfection with syphilis. A representative percentage of 14% reported having diabetes. 10% of the total respondents said they had disability, which is a percentage that is well below the national average of around 25%. Gastric (ulcer and gastritis) and liver (cirrhosis and hepatitis) problems appeared in 8.8% and 3.8%, respectively. Other infectious diseases such as mumps and Hansen’s disease had a prevalence of 3.8% of the participants. Two individuals pointed out having cancer and stroke, respectively.

Difficulties in accessing health services can substantially affect the identification and diagnosis of pre-existing diseases. In consequence, the appearance of tuberculosis and Hansen’s disease is a fact that should be considered by health boards.

Regarding COVID-19, the research shows that 6.9% of participants had symptoms related to this. However, many of the COVID-19 symptoms such as the flu, cough, fever, and tiredness are already part of the health issues experienced by those living on the streets. As a result of this, they may have gone unnoticed as something different from the usual for many people living in these conditions.

The low incidence of positive tests for COVID-19 among the sample does not necessarily indicate that they were protected against the virus. We know that access to health care services by this population is difficult, which may have resulted in undiagnosed cases. Other factors such as lack of documents and information on how and where to request a COVID-19 test may also have influenced the results. An important point to be considered is the low testing carried out in the country, which hinders epidemiological surveillance actions. It is also aggravating that there is no specification in the medical records of health care units to identify the access of this population group.

The difficulty in accessing health care services along with stigma and institutional racism is a crucial factor to increase the risk of complications and deaths by COVID-19. According to Pinho, Grando, and Pinho [27], the deprived conditions of homeless people are one of the manifestations of racism. Table 3 shows the impacts of COVID-19 in the participants of our research. The variables correspond to the different analysis categories.

## 4. Discussion

There are multiple causes of homelessness in Brazil. Escorel [28] (p. 139) says that “characters and scenarios of a social drama area naturalized in their misery and social isolation living on the streets without being able to settle and counting on their luck”. According to Silva [19], several factors lead to homelessness such as structural factors (lack of housing, lack of work and income, economic and institutional changes with a strong social impact, etc.), biographical factors (alcoholism, drug addiction, family relationship breakdown, mental illness, loss of all assets, etc.), in addition to the mass and/or natural disasters (floods, fires, etc.).

The prevalence of men within the homeless population is globally observed [29], and in Brazil, it is no different. Our research also showed that homelessness is a racial issue. The black population is particularly susceptible to this condition. Even in countries with a low number of black people as in the case of England, ethnic minorities and the black population are proportionally more vulnerable to be affected by homelessness, with less access to housing policies [30].

In Brazil, according to the Brazilian Institute of Geography and Statistics (IBGE), most of the Brazilian population is black or brown, corresponding to 55.8% of the total number of individuals. In the specific case of the homeless population, the National Survey revealed that 67% declared themselves black or brown, and 29.5% declared themselves white [7]. The Municipal Census also indicates that the prevalence of black people in the municipality of Rio de Janeiro is 79.6%. Following the same trend as the municipal and national census surveys, our sample also found that 85% of participants were men, with a high percentage of black population living on the streets (73% of the total).

Regarding the age group of the homeless population, we could not make a direct comparison between the data from the National Survey of 2008, the Municipal Census of 2018, and our survey, as the methodology is divergent. The National Survey showed a concentration between 25 and 44 years of age (53%). The Municipal Census indicates that most people are in the age group of 31 to 49 years. Our sample found out a prevalence of 33% among individuals aged 31 to 40 years and of 42% of people aged 41 to 59 years of age.

As seen, our research revealed a greater concentration of individuals of working age. In recent years, we have experienced the process of weakening of labor ties, unemployment, and loss of labor and social rights, as well the precariousness of work, which explains the high incidence of homeless people in the labor age group.

## 5. Conclusions

The COVID-19 pandemic is increasing poverty and impacting people’s lives in Brazil. There is a visible rise in the numbers of those experiencing homelessness and rough sleepers. The process of spreading the pandemic is deepening pre-existing inequalities, exacerbating vulnerabilities of the elderly people, those with pre-existing medical conditions, those who eat poorly, and those who have no home. While thousands of people are unemployed and have been thrown into poverty, the recovery for the world’s poorest people can take more than a decade, as the wealthiest, both individuals and businesses, thrive [31]. The virus exposed and increased inequalities in income, gender, and race, revealing how our deeply unequal, racist, and patriarchal system particularly affects women and black men and other racialized, excluded, and historically marginalized and oppressed groups in Brazil and the world. In our study, women were more impacted by COVIS-19 regarding health problems. Economic impacts were also felt more by women who were earning less and struggling to get ways of subsistence. Surprisingly, women participants in this study had high levels of education compared with men, which suggests that priority measures to ensure women’s equal representation in Government response for both the immediate response and longer-term recovery efforts are urgent and necessary.

The lack of official data on this population in Brazilian leads to reflect on how to carry out health surveillance when there is no specific record on information and knowledge about this population in most health information systems. The lack of general data on this population reproduces and reaffirms the social invisibility of these people, who are mostly brown and black bodies that become ill and die from various causes, and yet this does not seem to have an impact. Their deaths are naturalized by society [32].

Several testimonies reinforced the worsening of the situation of extreme vulnerability and poverty already experienced by these groups before the pandemic. However, due to the scarcity of means of subsistence on the streets during the period of social distancing, it became much worse, putting at risk the survival of these groups. The benefit offered by the Brazilian federal government for a short period to those unable to provide for themselves during the pandemic did not reach the hands of this population. Registration protocols to access the benefit as a fixed address and other basic documents that this homeless population could not provide prevented them from accessing a right to which they were entitled.

Regarding the measures to contain the transmission of the virus, most of the health recommendations on COVID-19 transmitted to society were not easily applicable to the daily lives of those who are homeless. Thus, the importance of articulated actions among the civil society, universities, and health and social care sectors to fight the contagion of COVID-19 in this population must be highlighted. The study showed the central role of non-governmental organizations in the creation of key strategies to control the spread of the virus in this population (such as the distribution of clean water and hygiene items for the population living on the streets), to minimize the financial impact on their income with the closed shops (distribution of daily meals) and to increase emotional support, and to provide information on how to protect themselves (groups of volunteers went to the streets to talk to the participants). This shows how the combination of emergency actions, health education, and intersectoral strategies has contributed to reducing the risk of contagion and illness by Covid-19 among people experiencing homelessness. Similar to any other member of society, this segment historically marked by invisibility is also entitled to the right to health before, during, and after the pandemic.

Respiratory problems were the health problem most mentioned by the study participants, and it becomes a concern for health sectors as the impact of COVID-19 for those who already have these problems usually brings severe complications, including the progression of respiratory problems. A recent report released by the State Health Secretariat of Rio de Janeiro on March 06, 2021 showed that 93% of the 616 Intense Care Unit places of the Unified Health System (SUS) available for patients with COVID-19 in the city of Rio de Janeiro were occupied [33]. Part of these places has been taken by patients coming from the north of the country, as the situation there is even more chaotic. This scenario is repeated in many other cities in Brazil. It reflects the increased number of infections and high numbers of daily deaths, which reveals the chaos that Brazil faces in the imminent saturation of hospital places for the treatment of COVID-19. This study has no statistical relevance for the entire homeless population of the city but may represent a first idea and key points to consider and further explore in broad studies.

To explore the social and health conditions affecting the homeless population aggravated due to the pandemic of COVID-19, the present study does not end with the information and data presented in this paper but helps us reflect on the challenges imposed by the pandemic in the expansion of the poverty and the inequalities’ aggravation resulting from it. The expressive and visible increase of people living on the streets in Rio de Janeiro due to the pandemic is notorious and was already being observed before the current lack of substantial investment by the Federal Government in the areas of health and social assistance. It requires the engagement of all sectors—public, private, non-governmental, and representatives of this population group—to build sustainable and viable alternatives for a better life for these people.

There is a lack of concrete actions to offer a more effective approach in tackling the pandemic and its health adversities for certain groups. Thus, as we expect the discussions around the world will focus on issues of equity, sustainability, and human dignity for those most vulnerable, we wish that the emphasis on a new political dimension with more social cohesion, solidarity, and social justice is on the horizon when responding to the COVID-19 crisis.

## Figures and Tables

**Table 1:** Profile of the Homeless Population.

Proportional Distribution of Homeless People According to the Sociodemographic Characteristics Based on the Sample Number (*n* = 304)		
Variables	f	%
Gender		
Female	45	14.8%
Male	259	85.2%
Race		
Black/brown	221	72.7%
White	69	22.7%
Yellow/Indigenous	1	0.7%
No answer	12	3.9%
Age group		
18 to 30 years	49	16.1%
31 to 40 years	99	32.6%
41 to 59 years	128	42.1%
Over 60	27	8.9%
No answer	1	0.3%
Nationality		
Brazilian	296	97.4%
Other	2	0.6%
No answer	6	2.0%
Education		
Literate	28	9.2%
Illiterate	23	7.6%
Complete high school	1	0.3%
Incomplete high school	52	17.1%
Complete elementary school	57	18.8%
Incomplete elementary school	130	42.8%
Complete higher education	2	0.6%
Incomplete higher education	6	2.0%
No answer	5	1.6%

Note: “f” represents the frequency that the responses appear in our sample. The “n” is related to the total number of participants.

**Table 2:** Access to Services.

Proportional Distribution of Homeless People Accessing the Services (304/f = %)		
Variables	f	%
Have you ever been approached by any of these services?		
Social care sectors	171	56.3%
Consultórios na Rua	47	15.5%
Security Agents	72	23.7%
Public Defender’s Office	27	8.9%
None	57	18.8%
No answer/Blank	11	3.6%
When you have a health problem, how do you treat it?		
Public services	244	80.3%
Third sector volunteers/churchs	30	9.9%
Never needed	3	1.0%
Self-care, seek the family, alternative care	5	1.6%
None	29	9.5%
No answer/Blank	8	2.6%
Which of these do you consider the best support in case of a health problem?		
Basic Health Clinics and the Family Health Clinics	96	31.6%
Emergency Care Units	91	29.9%
Hospitals	27	8.9%
Consultórios na Rua	23	7.6%
Third sector’ volunteers/churchs	31	10.2%
None	22	7.2%
No answer/Blank	14	4.6%

Note: “f” represents the frequency that the responses appear in our sample. The “n” is related to the total number of participants.

**Table 3:** Impacts of Covid-19.

Proportional Distribution of Homeless People, According to Experiences during the Pandemic (304/f = %)		
Variables	f	%
Hygiene items	155	51.0%
Conversation and emotional support	60	19.7%
Food (meals or breakfast)	253	83.2%
Food Baskets	31	10.2%
Social benefits	15	4.9%
Cothes and medicine	5	1.6%
None	5	1.6%
No answer/Blank	9	3.0%
Do you have pre-existing conditions?		
No	203	66.8%
Yes	80	26.3%
Do not know/Do not remember	18	5.9%
No answer/Blank	3	1.0%
If so, which ones?		
Heart and hypertension problems	22	27.5%
Respiratory problems	18	22.5%
HIV and STDs	14	17.5%
Diabetes	11	13.8%
Disabilities	8	10.0%
Gastric Problems	7	8.8%
Liver diseases	3	3.8%
Other infectious diseases	3	3.8%
Cancer and stroke	2	2.5%
Other	5	6.3%
Did you have COVID-19?		
I do not know; I did not feel any symptoms	277	91.1%
I felt some symptoms, but I was not diagnosed	21	6.9%
Yes, with confirmation exam	6	2.0%

Note: “f” represents the frequency that the responses appear in our sample. The “n” is related to the total number of participants.
